# A controlled study of community-based exercise training in patients with moderate COPD

**DOI:** 10.1186/1471-2466-14-125

**Published:** 2014-08-04

**Authors:** Shefalee Amin, Marlon Abrazado, Molly Quinn, Thomas W Storer, Chi-Hong Tseng, Christopher B Cooper

**Affiliations:** 1 Exercise Physiology Research Laboratory, Departments of Physiology and Medicine, David Geffen School of Medicine at University of California, Los Angeles, 37-131 Center for Health Sciences, 10833 Le Conte Avenue, Los Angeles, CA 90095-1690, USA; 2 Department of Biomathematics, David Geffen School of Medicine at UCLA, AV-327 Center of Health Sciences, 10833 Le Conte Avenue, Los Angeles, CA 90095-1690, USA

**Keywords:** Community, Exercise rehabilitation moderate COPD

## Abstract

**Background:**

The effectiveness of clinic-based pulmonary rehabilitation in advanced COPD is well established, but few data exist for less severe patients treated in alternative settings. The purpose of this study was to investigate whether a novel, community-based exercise program (CBE) was feasible and effective for patients with moderate COPD.

**Methods:**

Nineteen patients with moderate COPD (mean FEV_1_ 62%) and self-reported exercise impairment were randomized to 12-weeks of progressive endurance and strength training at a local health club under the guidance of a certified personal trainer, or to continuation of unsupervised habitual physical activity. Outcomes assessed at baseline and 12 weeks included session compliance, intensity adherence, treadmill endurance time, muscle strength, dyspnea, and health status.

**Results:**

Compliance was 94% and adherence was 83%. Comparisons between CBE and control groups yielded the following mean (SEM) differences in favor of CBE: endurance time 134 (74) seconds versus -59 (49) seconds (P = 0.041) and TDI 5.1 (0.8) versus -0.2 (0.5) (P < 0.001). The CBE group increased muscle strength (weight lifted) by 11.8 kilograms per subject per week of training (P < 0.001). SGRQ was not significantly changed.

**Conclusions:**

We demonstrated the feasibility and effectiveness of a novel community-based exercise program involving health clubs and personal trainers for patients with moderate COPD.

**Trial registration:**

ClinicalTrials.gov Identifier NCT01985529.

## Background

Chronic obstructive pulmonary disease (COPD) is a progressive debilitating disease that leads to worsening symptoms and declining exercise capacity [1]. Progressive weight loss is also common [2] and associated with increased mortality and compromised quality of life [3]. Skeletal muscle dysfunction is recognized to account for an appreciable portion of the exercise impairment in COPD patients [4]. Evidence for this particular abnormality includes reduced muscle aerobic enzyme content [5,6] and a tendency to develop premature lactic acidosis during exercise [1]. Furthermore, calf muscle cross-sectional area is reduced compared with controls [7] and muscle strength is also impaired [8].

Importantly, COPD is now regarded as preventable and treatable [9]. One could argue that successful treatment requires earlier recognition of the decline in exercise performance and implementation of a structured exercise program to reverse its deleterious effects.

Structured exercise training is strongly endorsed for COPD in the context of a pulmonary rehabilitation program recognizing that these programs increase functional capacity, decrease symptoms, reduce utilization of health-care resources and improve quality of life [10-12]. The Global Initiative on Obstructive Lung Disease (GOLD) recommends that patients with COPD should be referred for pulmonary rehabilitation once their FEV_1_ falls below normal [13]. This equates to the transition from mild to moderate disease and corresponds with the time when patients begin to significantly reduce activities of daily living [14]. Furthermore, the American Thoracic Society/European Respiratory Society Statement on Pulmonary Rehabilitation suggests all patients suffering from respiratory disease associated with diminished functional capacity and reduced health related quality of life may benefit from pulmonary rehabilitation [12].

While pulmonary rehabilitation is practiced as a multi-disciplinary therapy, evidence-based analysis identifies exercise training as the most effective component [15]. Despite guidelines, a national survey of 283 pulmonary rehabilitation programs in 1995 identified considerable differences in program content [16]. Lack of consistency in the approach to aerobic and resistance training in chronic pulmonary disease is likely to result in sub-optimal outcomes in many cases.

A paradigm shift is needed to make exercise training programs for COPD patients more scientifically rigorous and cost-effective. We have studied the feasibility of a community-based exercise program under the supervision of personal trainers appropriately certified in exercise physiology and trained to manage pulmonary patients safely and with confidence. We examined the hypothesis that in patients with moderate COPD and reduced aerobic capacity, the combination of aerobic exercise training plus nutritional counseling would result in improvement in exercise endurance, muscle strength, activity levels and quality of life compared with control group having only nutritional counseling. A secondary aim was to demonstrate the feasibility of community-based exercise rehabilitation delivered through health clubs and personal trainers at an earlier stage in the progression of COPD.

## Methods

### Subjects

We recruited 19 men and women with moderate COPD as defined by the GOLD [17] criteria (FEV_1_/FVC, % <70%; FEV_1_ < 70% and >50% predicted), a 10 pack-year smoking history and self-reported functional impairment. Exclusion criteria included current smokers, pulmonary diseases other than COPD, use of supplemental oxygen, musculoskeletal disease that impaired exercise performance and unstable coronary artery disease or congestive heart failure. The study was approved by the UCLA Office for the Protection of Research Subjects (IRB#11-000175) and all subjects gave written informed consent.

### Study design

**Figure 1:**
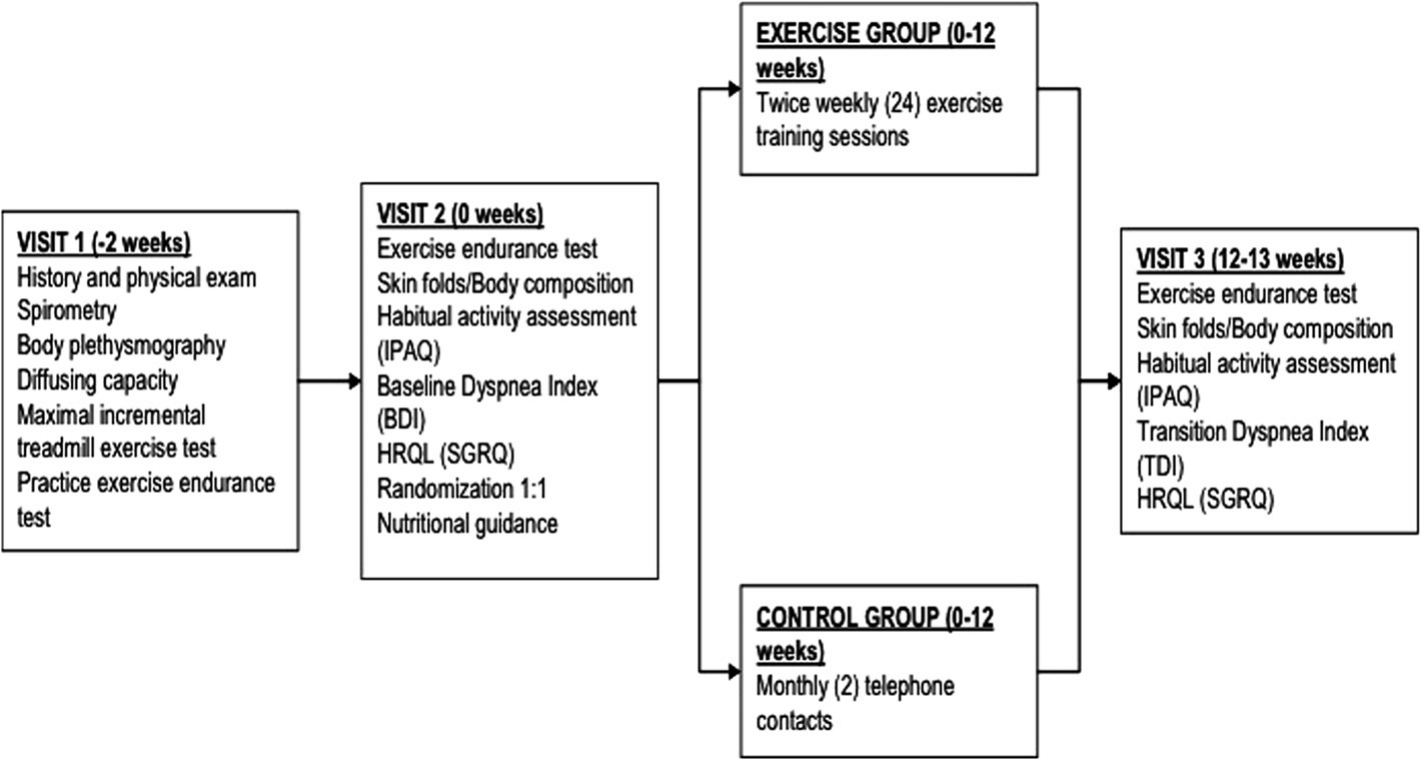
Study design showing time frame and procedures at each visit.

This was a small parallel-group, randomized controlled study to assess the feasibility of implementing a community-based exercise program for patients with moderate COPD (see Figure 1). At the screening visit, spirometry was performed to assess eligibility in terms of the GOLD staging followed by body plethysmography, diffusing capacity and a maximal, symptom-limited, incremental exercise test on a treadmill ergometer using a standardized protocol [18]. Then, after a resting period of 2 hours, they performed a constant work-rate exercise test at 90% of the maximum work rate calculated from the incremental test.

Within two weeks of the screening visit, subjects returned to repeat the constant work-rate exercise test also on a treadmill at the same constant work rate as at the first visit. This was intended to overcome any learning effect, and the best value for endurance time from the two constant load tests was used for future comparison. Subjects had assessments of body composition, dyspnea (Baseline Dyspnea Index, BDI) and quality of life (St. George’s Respiratory Questionnaire, SGRQ) [19,20].

Although not felt to be malnourished, all subjects were given nutritional guidance from a certified nutritionist by way of best clinical practice and also so as to be able to offer some benefit to those subjects randomized to the control group. The nutritional counseling was based on a Food Frequency Questionnaire [21] administered every two months to assess diet and specifically to ascertain the habitual protein intake of the subject. Detailed, written recommendations were then given on an individualized basis with the goal of achieving a daily protein intake of 1.5 g per kilogram of body weight plus a daily energy intake of 35 kilocalories per kilogram of body weight.

Subjects were then randomized 1:1 into two groups: (a) community based exercise program (CBE) or (b) usual habitual activity (control). Both groups continued standard COPD management based on published guidelines and appropriate for the stage of their disease. Subjects randomized to exercise training had measures of lower extremity strength by 1-repetition maximum (1-RM) for leg press at the time of the first training session. At 12 weeks, all patients were asked to repeat baseline measurements.

Minor exacerbations, defined as an increase in two or more symptoms (dyspnea, sputum volume and sputum purulence) for three or more days [22] but not requiring hospitalization, were referred to the subjects’ primary care physician for intensification of bronchodilator therapy and/or an antibiotic. Details of minor exacerbations were recorded but subjects continued in the study. Major exacerbations, requiring use of systemic corticosteroids or hospital admission, resulted in withdrawal from the study.

### Community-based exercise program

Following randomization, those subjects assigned to the exercise program were assigned to a personal trainer operating out of one of three local health clubs. They met at mutually convenient times, twice per week for 12 weeks. Each of the clubs provided facilities for aerobic exercise and resistance training. Two facilities provided two trainers each and a fifth trainer was in solo practice. All five trainers possessed Bachelors and/or Masters degrees in exercise science or related fields. Three were -Certified Strength and Conditioning Specialist (CSCS) from the National Strength and Conditioning Association and two held Exercise Specialist certifications from the American College of Sports Medicine. Specific methodologies were thoroughly reviewed with each personal trainer prior to the study along with general information regarding COPD and its effects on exercise tolerance and physical function. Initial, individualized aerobic and exercise prescriptions were developed for each subject based on findings from the maximal incremental exercise test performed at the screening visit. Information derived from this test was also used to define a safe ceiling for exercise intensity for each individual subject.

Each session included a warm-up and cool-down period. Subjects then performed 30 minutes of continuous aerobic exercise in the target heart rate range based upon an initial exercise prescription developed by the investigators. The trainers were entrusted with progressing the exercise prescription at reinforcement visits in order to maintain the appropriate exercise intensity. Subjects also performed resistance training designed upon basic principles, starting with one set of each of eight conventional free-weight or machine-based exercises for the large muscle groups of the upper and lower body and progressing to two sets (typically after 10-12 sessions) at the discretion of the personal trainer. The stipulated goal was to maintain 12 repetitions of each exercise in correct form before muscle fatigue (typically 70-75% of the maximal 1-RM weight).

Subjects assigned to the control group were told to continue their activities of daily living. They were contacted by telephone every month to see assess their progress.

### Outcomes

The co-primary outcome measures were endurance time for the constant work rate exercise test and change in muscle strength as calculated by total weight lifted per week. Secondary outcomes were lean body mass, percentage body fat, dyspnea (Transition Dyspnea Index, TDI) and SGRQ. Compliance was calculated as the percentage of sessions attended (out of 24), and adherence was calculated as the percentage of time subjects spent exercising in or above their target heart rate zone.

### Statistical analysis

The sample size for this study was estimated using an anticipated effect size for change in exercise endurance time derived from experience with bronchodilator therapy and other interventions in COPD patients. For example, acute administration of inhaled ipratropium bromide increased endurance time by 1.1-2.8 minutes and more recent work with the long-acting bronchodilator tiotropium bromide increased exercise endurance time for a similar work rate protocol by 105 seconds compared with placebo [23]. Importantly, changes in endurance time of this magnitude were felt to be associated with clinically meaningful perceptions of improvement on the part of the investigators and the patients. In a study of repeated constant work rate exercise testing in severe COPD patients (FEV_1_ = 40%) in clinically stable state, O’Donnell et al [24] showed that exercise endurance time was highly reproducible with a standard deviation of 60 seconds. From our database of submaximal CWR tests in moderate COPD patients repeated during the same week, we found a standard deviation for endurance time of 88 seconds. Our power analysis, based on a standard deviation of 88 seconds, α = 0.05 and 10 subjects in each group indicates a power of 86% (β = 0.14) to detect a 120 second difference in endurance time between the two groups after 3 months.

We used a two-sample *t*-test to evaluate whether exercise endurance time was longer in the subjects treated with exercise training and nutritional counseling versus nutritional counseling alone. We also carried out regression analysis to adjust for baseline covariates including age, gender and habitual activity level. Similar statistical methods as employed for the primary outcome measure were also applied to compare body mass index, TDI and SGRQ between groups.

## Results

Between August 2008 and December 2010, 29 patients were consented for the trial. Five patients failed screening. Twenty-four patients were randomized. Five patients in the exercise group withdrew from the study after randomization (one with worsening knee pain, one with an unrelated ankle injury, one with exacerbation of COPD, one with newly diagnosed lung cancer and one who was lost to follow-up). The baseline characteristics for both the control and exercise groups are listed in Table 1. The two groups were balanced in terms of age, anthropometrics, body composition, resting pulmonary function and breathlessness. The average FEV_1_ of all subjects was 62%, indicating moderate severity of COPD. Those randomized to the exercise group completed 202 of 216 total sessions, resulting in 94% compliance with the CBE. These subjects performed endurance exercise in or above the prescribed target heart rate zone 83% of the time.

**Table 1:** Baseline characteristics of the control and community-based exercise groups

	**Control group**	**CBE group**
No. of subjects	10	9
Age (year)	72.0 (10.1)	66.8 (8.1)
Male sex (%)	60	33
Height (m)	1.70 (0.1)	1.72 (0.1)
Weight (kg)	76.2 (10.9)	76.6 (23.4)
FEV_1_ (%)	60.8 (10.9)	63.6 (7.6)
Resting heart rate (/min)	82.7 (8.3)	76.4 (9.9)
O_2_ saturation (%)	96 (2)	96 (2)
BDI	6.5 (0.6)	6.6 (0.9)
Body fat (%)	27.1 (8.2)	32.5 (3.1)
Lean body mass (kg)	55.8 (10.4)	56.4 (16.7)

Values are mean (standard deviation). FEV_1_: forced expiratory volume in one second. O_2_ saturation: oxygen saturation. BDI: Baseline Dyspnea Index. m: meters. s: seconds. kg: kilograms.

**Figure 2:**
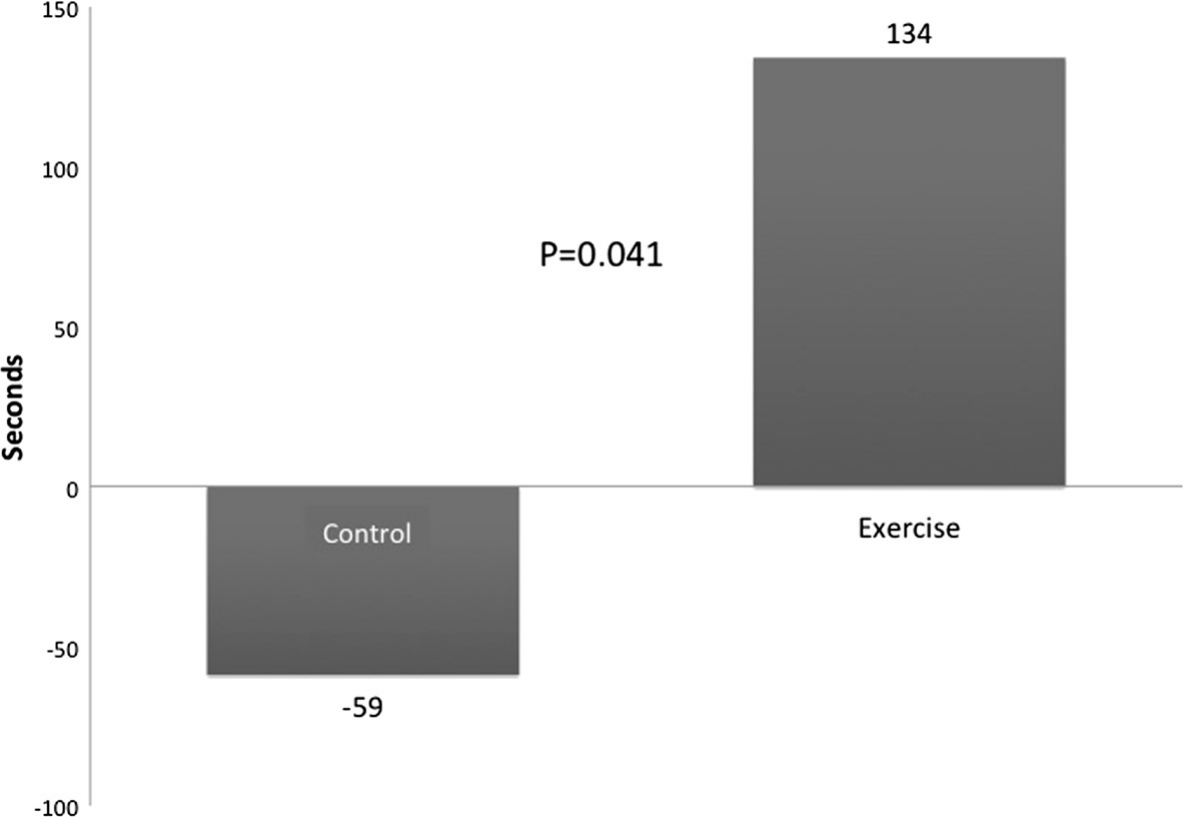
**Mean treadmill endurance times for the two groups at baseline and after 12 weeks.** Mean changes were a decrease of 59 seconds for the control group compared with an increase of 134 seconds for those completing the community-based exercise program (P = 0.041).

**Figure 3:**
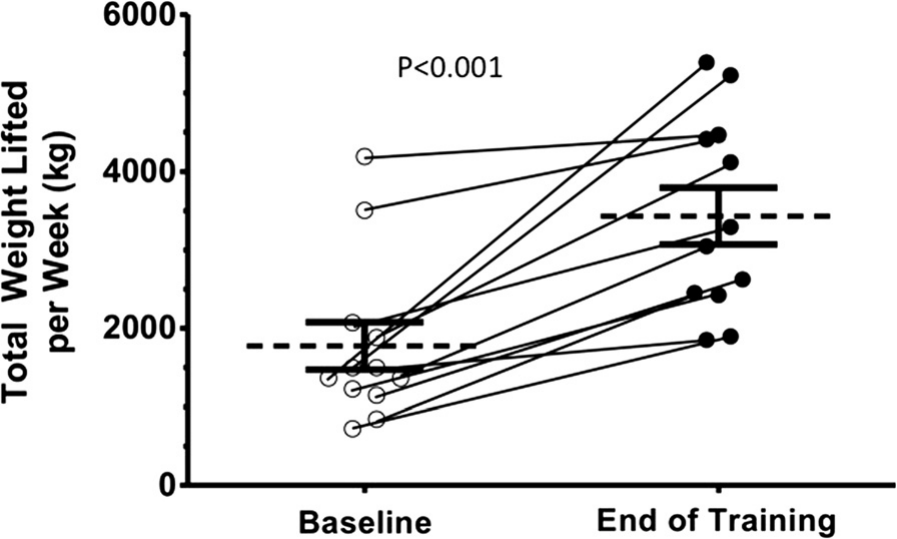
**Changes in total weight lifted for individual subjects from baseline to completion of training.** Total weight lifted per week is calculated as the product of load (kilograms) × repetitions × sets for two days in week one and two days at end of study. Diagonal lines represent individual changes. Horizontal lines represent group means and error bars are SEM (P < 0.001).

**Figure 4:**
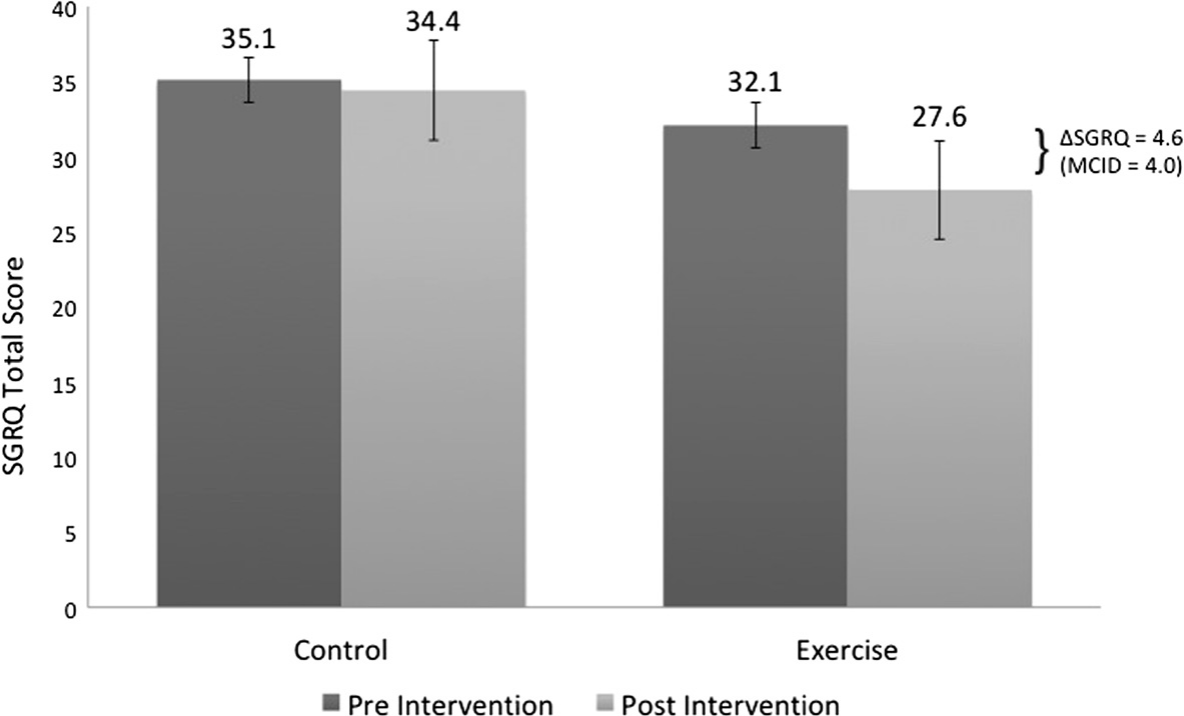
**Mean SGRQ scores for the two groups at baseline and after 12 weeks.** Mean changes were a decrease of 0.7 for the control group and a decrease of 4.6 for those completing the community-based exercise program (P = 0.611). Decreased scores represent improvement in quality of life where a change of 4.0 is considered clinically meaningful.

**Figure 5:**
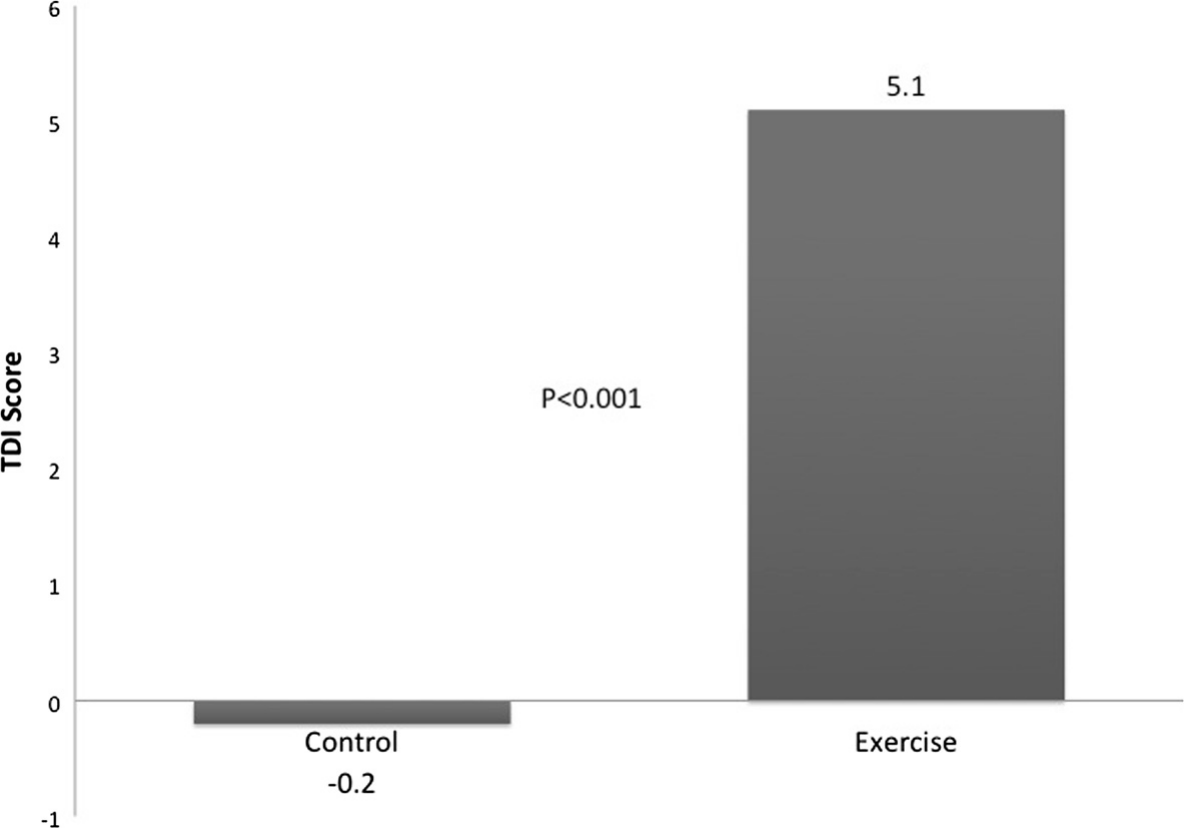
**Mean TDI focal score for the two groups after 12 weeks.** Mean changes were a decrease of 0.2 for the control group and an increase of 5.1 for those completing the community-based exercise program (P < 0.001). Increased scores represent improvement in dyspnea where a change of 1.0 is considered clinically meaningful.

As shown in Table 2 and Figure 2, at the end of 12 weeks, endurance time increased 134 seconds in the CBE group and decreased 59 seconds in the control group (P = 0.041). Patients who participated in the CBE arm lifted significantly more weight at the end of the exercise program compared with the beginning as shown in Figure 3: 3432 versus 1775 kilograms (P < 0.001). This represents a 93% overall improvement over 12 weeks, averaging an increase of 11.8 kilograms of weight lifted per subject for every week of exercise training. Figure 4 shows that SGRQ total scores decreased by 0.7 in the control group and 4.6 in the CBE group (P = 0.611). Whilst the differences between groups were not significant, the average fall in SGRQ total score within the exercise training group exceeds the MCID of 4 units which was not the case with the control group. TDI focal score, shown in Figure 5, increased by 5.1 in the CBE group versus a decrease of 0.2 in the control group, indicating less breathlessness (P < 0.001). There was no difference in body composition as determined by skin folds between the CBE and control group after intervention (change in body fat -1.1% versus -0.1%, P = 0.285; change in lean body mass +1.2 kilograms versus +0.1 kilograms, P = 0.206).

**Table 2:** Change in exercise performance, quality of life and dyspnea in control patients versus those in the community-based exercise program

	**Control group**			**CBE group**			**P-value**
	**Pre**	**Post**	**Change**	**Pre**	**Post**	**Change**	
Endurance time (s)	408 (67)	350 (89)	-59	444 (121)	578 (147)	+134	0.041
SGRQ total score	35.1 (3.8)	34.4 (6.2)	-0.7	32.1 (5.4)	27.6 (4.6)	-4.6	0.611
TDI focal score	NA	-0.2 (0.5)	-0.2	NA	5.1 (0.8)	+5.1	<0.001
Total weight lifted (kg/week)	NA	NA	NA	1775 (303)	3432 (361)	+1657	<0.001

Values are mean (SEM). SGRQ: St. George’s Respiratory Questionnaire. TDI: Transitional Dyspnea Index. s: seconds. Kg/wk: kilograms per week.

NA: not applicable.

## Discussion

We successfully conducted this small, randomized, feasibility study of a community-based exercise program versus habitual activity in patients with moderate COPD. We found increases in exercise endurance time and muscle strength, along with improved health-related quality of life, and reduced dyspnea in those who underwent exercise training compared to those who received nutritional counseling only. The average improvement in endurance time was 193 seconds at 90% of maximum work rate at baseline which compares favorably with changes in endurance time between 100 and 120 seconds in therapeutic trials of inhaled bronchodilators [23,25-27]. Furthermore, compliance and adherence to this community-based exercise program surpassed published rates for clinic- or hospital-based exercise programs [28].

We were unable to demonstrate a statistically significant and clinically meaningful difference in health status between the two groups as measured by SGRQ total score. There are likely two reasons for this: (1) with relatively small numbers of subjects the study was underpowered to prove the SGRQ changes statistically significant and (2) the difference between the groups was narrowed by a Hawthorne effect in the control group. Taking into account these shortcomings of our analysis, we feel that the reduction of SGRQ of -4.6 units in the training group is likely a real effect and brings our results, in terms of the effects of an exercise program on health status, into better alignment with the results of other studies.

Other studies on community based exercise programs have demonstrated improvements in functional capacity, dyspnea, and disease specific quality of life [29-32]. However, these studies have focused on patients with severe COPD (FEV_1_ < 50%) whereas our subjects were less severe (mean FEV_1_ 62%). We specifically sought to follow GOLD [33] and ATS/ERS [12] guidelines by recruiting patients with less severe disease. We reasoned that these patients would find a community-based exercise program, close to their home or workplace, more convenient than the typical clinic-based or hospital-based program. We also surmised that less severe patients would feel comfortable exercising in a health club environment alongside apparently normal individuals. The compliance with recommended training sessions of 99% attests to the success of this strategy. Furthermore, knowing that supervised exercise training is significantly more successful than unsupervised training, we utilized personal trainers to improve subject adherence to exercise prescriptions. The 83% adherence to the target heart rate range for aerobic training is impressive and clearly associated with marked improvements in the prespecified outcome measures of exercise performance.

We are aware of two other studies that examined outpatient rehabilitation in patients with moderate COPD. Cambach et al [34] found improved exercise tolerance and quality of life in patients with moderate COPD after 6 months of rehabilitation, however, this sample of patients also included asthmatics whose disease state might have been more fluctuant. Additionally, this rehabilitation program was conducted in various medical practices under the guidance of clinically trained physiotherapists, rather than in a local health club with certified personal trainers as in our community-based program. In another study, van Wetering, *et al*[35] randomized 199 patients with moderate COPD (mean FEV_1_ 60%) to an interdisciplinary community-based COPD management program (INTERCOM) or usual care. This study was conducted in the Netherlands where community-based physical therapists can be assigned to visit patients in their homes. The investigators reported improvements in health related quality of life, breathlessness, exercise performance, muscle strength, and body composition, but were unable to show improvements in muscle strength. Although this rehabilitation program was conducted in the community, it utilized clinically trained physical therapists and other medical personnel. Despite the promising data in favor of community based rehabilitation programs, it remains clear that there is not yet any consensus regarding the ideal structure for such programs. The traditional medical model of pulmonary rehabilitation is focussed on exercise encouragement in more severely disabled patients. The goals are often limited to tolerance of dyspnea and modest gains in exercise performance are considered acceptable. Our program is the first to use certified personal trainers, based in health clubs, which are becoming increasingly prevalent in the community. This health club model is more directed to maximizing performance using basic principles of reconditioning and muscle strengthening. Whilst there is undoubtedly considerable overlap in these approaches, we think that the personal trainers can potentially bring a new dimension to pulmonary rehabilitation.

The major limitation of our study was its small size. However, this was a pilot study and one of the main purposes was to demonstrate the feasibility of CBE training in moderate COPD. Our results should prompt further research to standardize procedures and define optimal interventions. A further limitation is that, although subjects were randomly assigned to two groups (exercise training or usual physical activities), the individual subjects and the trainers obviously could not be blinded to their treatment allocation. We attempted to reduce observer bias by having the baseline and final assessments performed in the research laboratory, distant from the training location, with the technician blinded and specifically instructed not to inquire about group allocation. Our exercise program was novel, but there was incomplete standardization of the exercise facilities involved in the study and the variability in the training and experience of the personal coaches. The exercise facilities included a nationally distributed chain, a small privately-owned facility, and a large privately-owned facility. The exercise coaches were all certified personal trainers and they received an educational session and information on exercise limitations in COPD patients. A future study might attempt to standardize the approach to training even further.

## Conclusions

In summary, our data confirm that community based exercise programs are not only feasible, but also effective, in patients with moderate of COPD. Such programs improve functional capacity, dyspnea, and health related quality of life, and should therefore be considered in the routine management of these patients. Furthermore, our findings contribute to a growing body of evidence that calls for a paradigm shift in the philosophy of pulmonary rehabilitation. As a treatment strategy, community-based exercise programs should be introduced earlier in the progression of COPD so as to forestall the development of comorbidities associated with the lack of regular exercise and deconditioning. Future efforts should be made to establish uniform guidelines to ensure that community-based exercise training programs for COPD patients are scientifically rigorous and cost-effective.

## Abbreviations

COPD: Chronic obstructive pulmonary disease; GOLD: Global initiative on obstructive lung disease; FEV_1_: Forced expiratory volume in one second; FVC: Forced vital capacity; BDI: Baseline dyspnea index; SGRQ: St. George’s Respiratory Questionnaire; CBE: Community-based exercise; 1-RM: 1-repetition maximum; TDI: Transition dyspnea index; ATS/ERS: American Thoracic Society/European Respiratory Society; INTERCOM: Interdisciplinary Community-based COPD Management Program; SEM: Standard error of the mean.

## Competing interests

The community-based exercise training program was implemented at Equinox Fitness Club, Century City, CA. TWS and CBC serve as an uncompensated members of the Equinox Health Advisory Board. None of the other authors have any financial or non-financial competing interests.

## Authors’ contributions

SA drafted the manuscript. MA was involved in subject testing, data collection and analysis. MQ was involved in data collection. TWS was involved in the study design, assisted with data collection, and participated in manuscript preparation. C-HT performed the statistical analysis. CBC conceived the study, was involved in its design, assisted with data analysis and participated in manuscript preparation. All authors read and approved the final manuscript.

## Pre-publication history

The pre-publication history for this paper can be accessed here:

http://www.biomedcentral.com/1471-2466/14/125/prepub
